# Noncoding RNAs: the shot callers in tumor immune escape

**DOI:** 10.1038/s41392-020-0194-y

**Published:** 2020-06-19

**Authors:** Lei Liu, Qin Wang, Zhilin Qiu, Yujuan Kang, Jiena Liu, Shipeng Ning, Yanling Yin, Da Pang, Shouping Xu

**Affiliations:** 1 Department of Breast Surgery, Harbin Medical University Cancer Hospital, Harbin, 150081 China; 2 Heilongjiang Academy of Medical Sciences, Harbin, 150086 China

**Keywords:** Tumour immunology, Non-coding RNAs

## Abstract

Immunotherapy, designed to exploit the functions of the host immune system against tumors, has shown considerable potential against several malignancies. However, the utility of immunotherapy is heavily limited due to the low response rate and various side effects in the clinical setting. Immune escape of tumor cells may be a critical reason for such low response rates. Noncoding RNAs (ncRNAs) have been identified as key regulatory factors in tumors and the immune system. Consequently, ncRNAs show promise as targets to improve the efficacy of immunotherapy in tumors. However, the relationship between ncRNAs and tumor immune escape (TIE) has not yet been comprehensively summarized. In this review, we provide a detailed account of the current knowledge on ncRNAs associated with TIE and their potential roles in tumor growth and survival mechanisms. This review bridges the gap between ncRNAs and TIE and broadens our understanding of their relationship, providing new insights and strategies to improve immunotherapy response rates by specifically targeting the ncRNAs involved in TIE.

## Introduction

According to the immunoediting theory, immune escape is the key to tumor survival.^1^ There are many mechanisms of tumor immune escape (TIE), including defects in tumor antigen presentation to escape recognition by the immune system, alterations in the tumor death pathways to achieve increased resistance to cytotoxic immune responses, metabolic alterations to promote TIE, and acquisition of stem cell-like phenotypes to escape immune-based recognition and destruction. In addition, some cytokines in the tumor microenvironment (TME), abnormal expression of immune checkpoint molecules on tumor or immune cell surfaces, and some immunosuppressive cells are all involved in TIE. Collectively, these factors may enable TIE, leading to a low response rate to immunotherapy in different malignancies.

Noncoding RNAs (ncRNAs), which cannot be translated into proteins, comprise 98% of the transcriptome. Generally, ncRNAs less than 50 nucleotides in length are defined as small ncRNAs (sncRNAs), including microRNAs (miRNAs), Piwi-interacting RNAs (piRNAs), transfer RNAs (tRNAs), small nuclear RNAs (snRNAs) and small interfering RNAs (siRNAs).^2^ A recent study reported the presence of partial sncRNAs derived from tRNAs, such as tRNA halves (tiRNAs) and tRNA fragments (tRFs).^3^ ncRNAs with more than 200 nucleotides are defined as long ncRNAs (lncRNAs), including long or large intergenic ncRNAs (lincRNAs), some circular RNAs (circRNAs), and ribosomal RNAs (rRNAs).^4^ The biological functions of ncRNAs, such as regulating gene expression at the transcriptional and translational levels, guiding DNA synthesis or gene rearrangement, and protecting the genome from foreign nucleic acids, have been gradually elucidated.^5^ An increasing number of studies indicate that ncRNAs are indispensable in tumorigenesis by regulating the expression of tumor-related genes. Mechanistically, lncRNAs regulate gene expression mainly by acting as transcription factors, regulating chromatin remodeling, or participating in posttranscriptional regulation as ceRNAs.^6^ circRNAs can regulate gene expression at epigenetic, transcriptional, and posttranscriptional levels (primarily as ceRNAs).^7^ miRNAs mostly regulate gene expression at the posttranscriptional level through RNA interference by binding to the 3′-untranslated region (3′UTR) (rarely 5′UTR or coding sequence) of protein-coding mRNAs.^8–10^ In addition, some TRFs and tiRNAs can participate in gene regulation and gene silencing via complementary binding with target genes, and the mechanism is similar to that of miRNA.^11^

Currently, ncRNAs involved in TIE are gradually emerging and are promising potential targets of antitumor therapy. Several studies have reported that ncRNAs play pivotal roles in TIE.^12,13^ Therefore, it is essential to systematically elaborate the complex regulatory network of TIE regulated by ncRNAs. In this review, we provide a detailed account of the molecular regulatory mechanisms underlying ncRNA involvement in TIE. We hope this review will broaden our understanding of the relationship between ncRNAs and TIE and provide new insights to target ncRNAs in TIE-associated therapeutic strategies.

## Defective antigen presentation and TIE

**Fig. 1:**
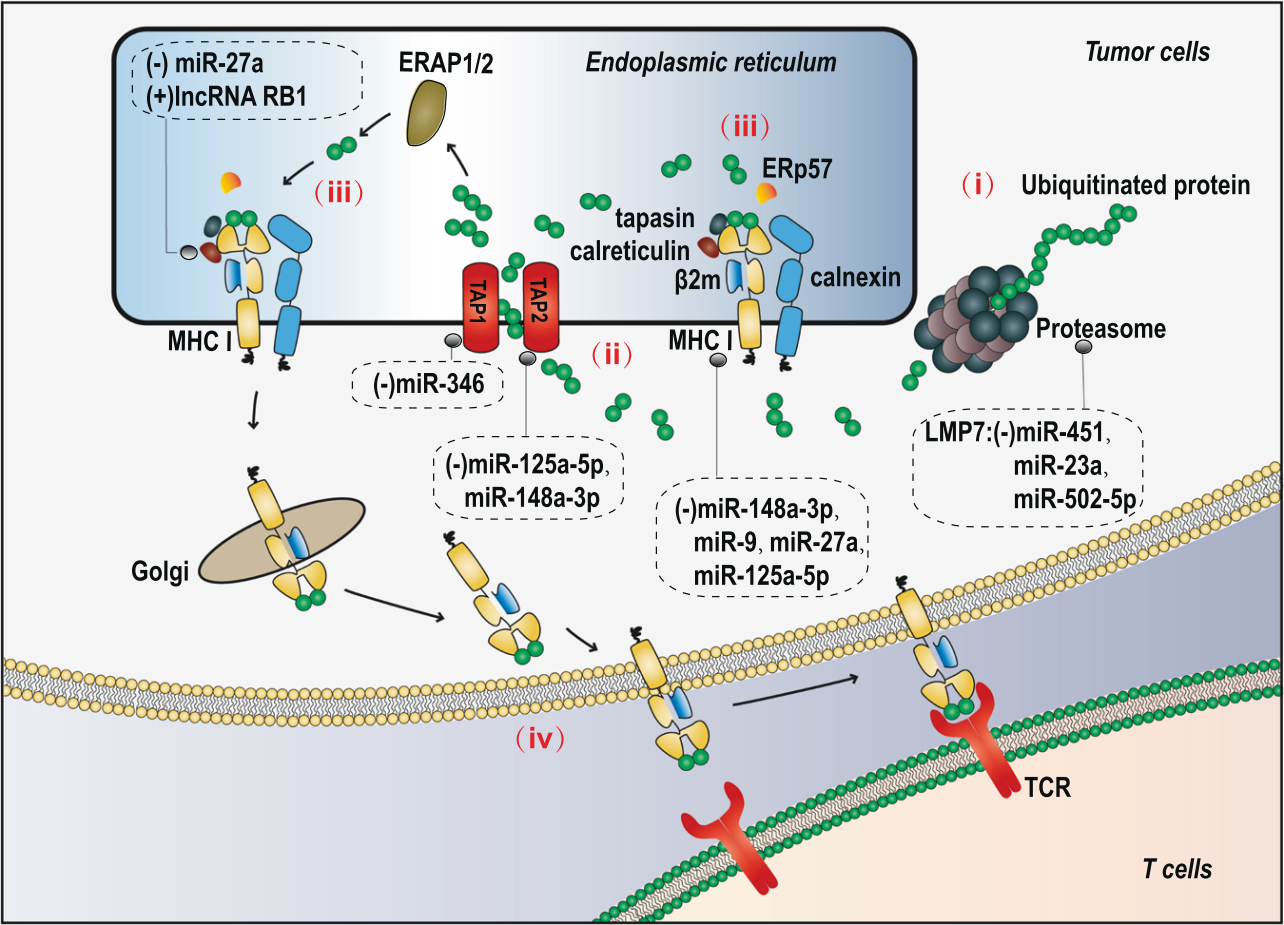
Regulation of ncRNAs in the four steps of MHC class Ι molecule-mediated APM

Tumor cells can achieve TIE by inhibiting antigen presentation, which enables T cells and/or natural killer (NK) cells to recognize and destroy target tumor cells. The regulation of the antigen processing and presenting machinery (APM) in tumor cells is dependent on the ubiquitin-protease system and the major histocompatibility complex (MHC) class Ι molecules. The mediation of the APM by MHC class Ι molecules is divided into four steps: (i) peptide generation and modification; (ii) peptide transport; (iii) assembly of the peptide-MHC class I complex; and (iv) antigen presentation (Fig. 1). ncRNAs have been reported to regulate the APM in tumors during these four steps. First, peptide generation and modification can be inhibited by low molecular mass protein 2 (LMP2), LMP7, and LMP10 in tumor cells.^14–16^ In liver cancer^17^ and gastric cancer,^18^ miR-23a and miR-502-5p, respectively, can inhibit the expression of LMP7 by interfering with interferon regulatory factor-1 (IRF-1), which is an essential factor in the INF-γ-mediated increase in LMP7;^19^ miR-451 has also been found to be able to directly regulate LMP7 in diabetic nephropathy,^20^ but whether such a regulatory relationship exists in tumors remains to be studied. Next, the expression of transporters associated with antigen processing (TAP) is blocked in tumors, thereby preventing peptide transport to the endoplasmic reticulum.^15,21^ miR-125a-5p and miR-148a-3p can reduce the levels of TAP2 and MHC molecules by binding to the 3′UTR of TAP2 mRNA in esophageal adenocarcinoma.^22^ miR-346 has been shown to bind to the 3′UTR of TAP1 mRNA and decrease TAP1 expression.^23^ Moreover, formation of the peptide-MHC class I complex is prevented via suppression of the expression of chaperone proteins (calnexin, ERp57, calreticulin, and tapasin) and the subsequent loading of peptides on MHC class I molecules.^21^ lncRNA RB1 can positively regulate calreticulin in multiple tumor cell lines.^24^ miR-27a has been found to downregulate the expression of calreticulin and MHC class I molecules in colorectal cancer, and the infiltration and cytotoxic activity of CD8^+^ T cells (the release of perforin) are inhibited.^25^ Furthermore, defective surface expression of MHC class I molecules, which would repress the APM, has been shown in other tumors.^26–28^ miR-27a, miR-148a-3p, miR-125a-5p and miR-9 have been found to inhibit the surface expression of MHC class I molecules in colorectal cancer, esophageal adenocarcinoma and nasopharyngeal carcinoma.^22,25,29^ ncRNAs participating in the inhibition of tumor antigen presentation by repressing key proteins provide direct evidence that these ncRNAs play important roles in the development of TIE. Interfering with the roles of ncRNAs in tumor antigen presentation may provide a new direction for improving the effect of tumor immunotherapy.

## Tumor death pathways and TIE

By changing the balance between pro-death signals and anti-death signals, tumor cells can gain resistance to cytotoxic immune responses and thus achieve TIE. First, increased expression of various antiapoptotic proteins (BCL-2, BCL-xL, and MCL-1) in tumor cells can enhance the apoptotic resistance of tumor cells.^30–32^ Second, inhibition of the expression of apoptosis-related receptors (FAS, DR4, and DR5) and ligands (FASL and TRAIL) can also enable tumor cells to escape apoptotic pathway-induced cell death.^33,34^ Third, tumor cells can escape cytotoxic T lymphocyte (CTL)- and NK cell-mediated death by blocking the perforin/granzyme pathway in immune cells. For example, tumor cells can escape CTL-mediated cytotoxicity by overexpressing inhibitors of the perforin/granzyme pathway.^35^ Moreover, one study identified a receptor that acts as a decoy ligand, thereby protecting tumor cells from apoptosis.^36^

Several studies have confirmed that ncRNAs can assist tumors in achieving TIE by regulating the abovementioned molecules and proteins, which could disrupt the balance between anti-death and pro-death signals (Table 1). For example, miR-195, miR-24-2 and miR-365 can downregulate the expression of Bcl-2 and promote the apoptosis of tumor cells in breast cancer,^37^ while miR-125b and miR-106a can upregulate the expression of Bcl-2 to inhibit the apoptosis of leukemia cells and breast cancer cells and promote their proliferation and infiltration.^38,39^ miR-133a targets and inhibits the expression of Bcl-xL and Mcl-1, promoting apoptosis in osteosarcoma cells.^40^ Similarly, miR-25 inhibits DR4 expression in cholangiocarcinoma cells, thereby enabling these cells to escape apoptosis induced via TNF-related apoptosis-inducing ligand (TRAIL).^41^ In addition, lncRNA MAGI2-AS3 upregulates the expression of FAS and FASL and promotes apoptosis in breast cancer cells.^42^ In CTLs extracted from the pleural effusion of lung cancer patients, miR-23a was highly expressed and could inhibit the antitumor ability of CTLs by repressing the expression of granzyme B.^43^ These ncRNAs regulate the expression of death signal-related molecules and further help tumors achieve immune escape. Targeting these ncRNAs to reduce the interference of death signals may be of great significance to improve the efficiency of antitumor therapy.

**Table 1:** ncRNAs influence TIE via regulating tumor cells death signals by targeting death-related genes

ncRNAs	Target genes and function	Type of cancer	Refs.
miR-195, 24-2 and 365	Downregulate Bcl-2 and promote apoptosis of tumor cells	Breast cancer	^37^
miR-125b, miR-106a	Upregulate Bcl-2 and inhibit apoptosis of tumor cells	Leukemia, Breast cancer	^38,39^
miR-133a	Downregulates Bcl-xL and McL-1, and promotes apoptosis of tumor cells	Osteosarcoma	^40^
lncRNA HELH	Upregulates Bcl-xL, which mediated by miR-939, and inhibits apoptosis of tumor cells	Colorectal Cancer	^288^
lncRNA ASNR	Inhibits the degradation of Bcl-2 by targeting AUF1, and inhibits apoptosis of tumor cells	Stomach cancer, Colon cancer, Liver cancer, Lung cancer	^289^
lncRNA OPI5-AS1	Upregulates Bcl-2, which mediated by miR-448, and inhibits apoptosis of tumor cells	Lung adenocarcinoma	^290^
miR-125a-5p, 26a, 193b, 363, 101, 29a, 29b, 106a, 181b, 302b and 320	Downregulate MCL-1 and promote apoptosis of tumor cells	Colon cancer, Breast cancer, lymphomas, Multiple myeloma, Ovarian cancer, Acute myeloid leukemia, HCC, Cervical cancer	^291–301^
miR-205,133b and 218	upregulate MCL-1 and inhibit apoptosis of tumor cells	Lung cancer	^302,303^
miR-25	Downregulates DR4 and promotes apoptosis of tumor cells	Cholangiocarcinoma	^41^
miR-133b	Increases the sensitivity of tumor cells to TRAIL- mediated apoptosis by targeting FAIM	PC-3 and HeLa cell lines	^304^
miR-942	Reduces the sensitivity of tumor cells to TRAIL-mediated apoptosis by targeting ISG12a	Hepatocellular carcinoma, Gastric cancer	^305^
miR-221 and 222	Reduce the sensitivity to TRAIL-mediated apoptosis by targeting p27kip1	NSCLC	^306^
miR-212	Reduces the sensitivity of tumor cells to TRAIL-induced apoptosis by targeting PED	NSCLC, Liver cancer	^307^
miR-130a	Enhances the sensitivity of tumor cells to TRAIL-induced apoptosis by targeting MET	Lung cancer	^308^
miR-145, 216, 182 and 96	Reduce the sensitivity of tumor cells to TRAIL-induced apoptosis by targeting DR4/5, FADD	Breast cancer	^309^
miR-200c	Reduces the sensitivity of tumor cells to FAS-mediated apoptosis by targeting FAP-1	Human kidney clear cell cancer	^310^
miR-21	Downregulates FASL and inhibits apoptosis of tumor cells	Pancreatic cancer	^311^
miR-590 and 20a	Downregulate FASL and FAS, and inhibit apoptosis of tumor cells	Osteosarcoma	^312^
miR-128a	Downregulates FAS and inhibits apoptosis of tumor cells	Acute T-cell leukemia	^313^
lncRNA MAGI2-AS3	Upregulates FASL and FAS, and inhibits apoptosis of tumor cells	Breast cancer	^42^
miR-23a	Downregulates granzyme B and inhibits CTL-mediated death	Lung cancer	^43^
miR-27a	Downregulates granzyme B and perforin, inhibits CTL-mediated death	Colorectal cancer cell line sw260	^314^

*AUF1* ARE/poly (U)-binding/degradation factor 1, *FAIM* Fas apoptosis inhibitory molecule, *ISG12a* interferon stimulated gene 12a, *PED* PED/PEA-15, *FADD* Fas-associated death domain, *FAP-1* Fas-associated phosphatase-1, *NSCLC* non-small cell lung cancer

## Abnormal metabolism and TIE

### Aerobic glycolysis and TIE

Owing to mitochondrial dysfunction and despite being in an aerobic environment, tumor cells prefer to produce energy through glycolysis, which is accompanied by the production of a large amount of lactate. Such aerobic glycolysis phenomenon is termed the Warburg effect.^44^ This particular mode of metabolism provides the energy and macromolecules essential for the rapid growth and invasion of tumor cells. Lactate produced by aerobic glycolysis acidifies the TME, which can lead to the dysfunction of immune cells [cytotoxic T cells, dendritic cells (DCs), NK cells, and macrophages] and inhibit the secretion and function of several antitumor response cytokines. These alterations can subsequently lead to immunosuppression and promote tumor cells to escape destruction by the immune system.^45,46^ An increasing number of studies have found that ncRNAs can regulate tumor aerobic glycolysis directly (by targeting enzymes related to aerobic glycolysis) or indirectly (by targeting HIF-1α or tricarboxylic acid cycle (TAC)-related enzymes) to help tumors achieve TIE.

**Fig. 2:**
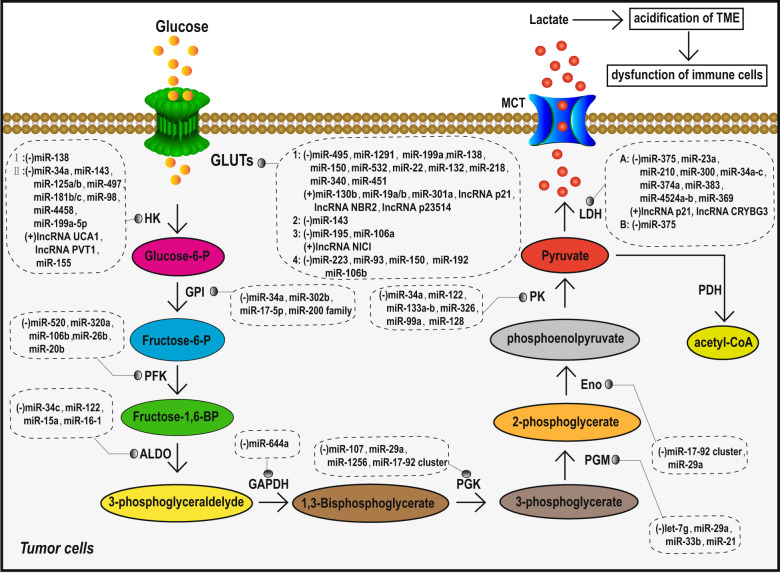
Overview of the regulation of TIE-associated glycolytic enzymes by ncRNAs in tumor cells

Glucose transporters (GLUTs) are membrane proteins that transport glucose into cells. Abnormal GLUT expression on the tumor cell surface promotes glucose transport into the cell and increases aerobic glycolysis. ncRNAs have been found to be involved in the regulation of GLUTs in human cancers (Table 2, Fig. 2). For example, miR-340, miR-1291, miR-495, miR-22, and miR-132 downregulate GLUT1 expression in various tumors,^47–51^ whereas miR-130b, miR-301a, miR-19a/b, lncRNA p23154, lncRNA NBR2, and lncRNA p21 promote GLUT1 expression.^52–55^ miR-150 and miR-195-5p downregulation promotes GLUT4 and GLUT3 expression in pancreatic cancer and bladder cancer, respectively.^56,57^

**Table 2:** ncRNAs influence TIE via regulating abnormal metabolism of tumor by targeting key enzymes

Target genes	ncRNAs	Function	Refs.
*Aerobic glycolysis*			
GLUT1	miR-495, 1291, 199a, 138, 150, 532, 22, 132, 218, 340 and 451	Downregulate GLUT1 and inhibit aerobic glycolysis	^47–51,315–317^
	miR-130b, 301a, 19a/b, lncRNA P21, lncRNA NBR2, lncRNA p23514	Upregulate GLUT1 and promote aerobic glycolysis	^52–55^
GLUT2	miR-143	Downregulates GLUT2 and inhibits aerobic glycolysis	^318^
GLUT3	miR-195 and miR-106a	Downregulate GLUT3 and inhibit aerobic glycolysis	^57,319^
	lncRNA NICI	Upregulates GLUT3 and promotes aerobic glycolysis	^320^
GLUT4	miR-223, 93, 150, 192 and 106b	Downregulate GLUT4 and inhibit aerobic glycolysis	^321–324^
HK1	miR-138	Downregulates HK1 and inhibits aerobic glycolysis	^59^
HK2	miR-34a, 143, 125a/b, 497, 181b/c, 98, 4458 and 199a-5p	Downregulate HK2 and inhibit aerobic glycolysis	^62,325–332^
	miR-155, lncRNA PVT1 and lncRNA UCA1	Upregulate HK2 and promote aerobic glycolysis	^60,61,327^
GPI	miR-34a, 302b, 17-5p and 200 family	Downregulate GPI and inhibit aerobic glycolysis	^333–335^
PFK	miR-520, 320a, 106b, 26b and 20b	Downregulate PFK and inhibit aerobic glycolysis	^336–341^
Aldo	miR-34c, 122, 15a and 16-1	Downregulate Aldo and inhibit aerobic glycolysis	^342–345^
GAPDH	miR-644a	Downregulates GAPDH and inhibits aerobic glycolysis	^346^
PGK	miR-107, 29a, 1256 and 17-92 cluster	Downregulate PGK and inhibit aerobic glycolysis	^77,347–349^
PGM	let-7g, miR-29a, 33b and 21	Downregulate PGM and inhibit aerobic glycolysis	^340,349–351^
Eno	miR-17-92 cluster and miR-29a	Downregulate Eno and inhibit aerobic glycolysis	^348,349^
PK	miR-34a, 122, 133a-b, 326, 99a and 128	Downregulate PK and inhibit aerobic glycolysis	^64,333,352–354^
LDHA	miR-375, 23a, 210, 300, 34a-c, 374a, 383, 4524a-b and 369	Downregulate LDHA and inhibit aerobic glycolysis	^65,66,355–360^
	lncRNA p21 and lncRNA CRYBG3	Upregulate LDHA and promote aerobic glycolysis	^67,361^
LDHB	miR-375	Downregulates LDHB and inhibits aerobic glycolysis	^65^
IDH	miR-183	Downregulates IDH, inhibits TAC and promotes aerobic glycolysis	^70^
SDH	miR-210	Downregulates SDH, inhibits TAC and promotes aerobic glycolysis	^71^
Cytochrome c oxidase 1 / 2	miR-181c and miR-338	Downregulate Cytochrome c oxidase 1 / 2, inhibit electron transport links and promotes aerobic glycolysis	^72,73^
HIF-1α	miR-17-92 cluster, 22, 33a, 107, 128, 138, 155, 186, 195, 516c and circEPHB4	Downregulate HIF-1α and inhibit aerobic glycolysis	^78–80,269,270,348,354,362–369^
	lncRNA SNHG1, 00152, DANCR, miR-21 and circRNA PIP5KA	Upregulate HIF-1α and promote aerobic glycolysis	^81,83,370,371^
*Arachidonic acid metabolism*			
PGE2	miR-206	Downregulates PGE2 and inhibits arachidonic acid metabolism of tumor	^88^
COX-2	miR-128, 146a, 101 and 143	Downregulate COX-2 and inhibit arachidonic acid metabolism of tumor	^89–92^
*Tryptophan metabolism*			
IDO	miR-153-3p	Downregulates IDO and inhibits tryptophan metabolism of tumor	^98^
	lncRNA SNHG1, lncRNA MALAT1,	Upregulate IDO and promote tryptophan metabolism of tumor	^96,97^

Several enzymes, such as hexokinase (HK), aldose enzyme, glucose phosphate isomerase (GPI), phosphofructokinase (PFK), aldolase (Aldo), glyceraldehyde-3-phosphate dehydrogenase (GAPDH), phosphoglycerate mutase (PGM), enolase (Eno), pyruvate kinase (PK), pyruvate dehydrogenase (PDH), and lactate dehydrogenase (LDH), are involved in glycolysis reactions. Abnormal expression of ncRNAs has been reported to alter the expression of these enzymes, thus accelerating the process of tumor glycolysis (Table 2, Fig. 2). miR-138 and miR-143 were found to regulate aerobic glycolysis in different types of tumor cells by directly targeting HK1 and HK2, respectively.^58,59^ miR-155 and lncRNA urothelial cancer associated 1 (lncRNA UCA1) can increase HK2 expression by inhibiting miR-143 in breast cancer and bladder cancer, respectively.^60,61^ In liver cancer, miR-199a-5p can directly target HK2 and repress HK2 expression to inhibit glycolysis.^62^ Moreover, miR-122 targets the aldose enzyme in liver cells,^63^ and miR-326 downregulation is associated with increased PK2 expression in glioblastoma cells,^64^ both of which can inhibit aerobic glycolysis of tumor cells. PDH is the key enzyme that catalyzes the conversion of pyruvate to acetyl-CoA. Under conditions of aerobic glycolysis in tumor cells, such conversion of pyruvate to acetyl-CoA is inhibited, and pyruvate is converted to lactate by LDH, thus promoting TME acidification and TIE. LDHB is regulated by miR-375 and is increased in esophageal squamous cell carcinoma,^65^ whereas LDHA, which is also overexpressed in tumor cells, is regulated by miR-34a, miR-34c, miR-369-3p, miR-374a, and miR-4524a/b in colorectal cancer, leading to more lactate production.^66^ lncRNA p21 positively regulates LDHA, pyruvate dehydrogenase kinase 4 (PDK4), pyruvate dehydrogenase complex (PDHX), PK2, and GPI simultaneously in different tumor cells to promote aerobic glycolysis and lactate production.^67^ PDH inhibition can reduce the tricarboxylic acid (TCA) cycle, thus promoting the conversion of pyruvate into lactic acid. miR-23a, miR-375, and miR-138-1^*^ upregulate PDH expression by inhibiting PDK, which is a negative regulator of PDH.^68,69^

In most tumor cells, the TAC is inhibited, which results in pyruvate being unable to be metabolized through TAC and only converted to lactic acid, exacerbating the acidification of TME. miRNAs can target and inhibit enzymes involved in the TCA cycle and several components of the electron transport chain, thereby inhibiting mitochondrial function and further promoting aerobic glycolysis (Table 2). For example, miR-183^70^ and miR-210^71^ can, respectively, target key enzymes isocitrate dehydrogenase (IDH) and succinic acid dehydrogenase (SDH) of the TCA cycle in glioma and lung cancer. miR-181c^72^ and miR-338^73^ downregulate cytochrome c oxidase 1/2, respectively, which are components of the electron transport chain.

Recent studies demonstrated that hypoxia-inducible factor-1α (HIF-1α) can promote aerobic glycolysis in tumor cells^74^ and then promote TME acidification and TIE. First, activated HIF-1α can directly or indirectly increase the expression of all glycolysis-related enzymes and promote glycolysis.^75^ Second, HIF-1α prevents the conversion of pyruvate to acetyl-CoA, the raw material of the TCA cycle, by inhibiting PDH activity.^74,75^ Moreover, HIF-1α indirectly promotes glycolysis by inhibiting mitochondrial oxidative phosphorylation.^76^ ncRNAs have been found to regulate HIF-1α in tumors, which could promote aerobic glycolysis and TIE (Table 2). For example, the miR-17-92 cluster,^77^ miR-22,^78^ miR-33a,^79^ and circRNA EPHB4^80^ have been reported to downregulate HIF-1α expression in different types of tumors, while miR-21 can promote the expression of HIF-1α in prostate cancer cells; lncRNA SNHG1,^81^ lncRNA 00152^82^ and circRNA PIP5KA^83^ function as molecular sponges for miR-18a, miR-138 and miR-600, respectively, to promote HIF-1α expression in different tumors. It has not been reported that ncRNAs and HIF-1α can directly affect the immune escape of tumor cells through complex molecular networks, but this indirect evidence also suggests that this idea merits further exploration.

### Arachidonic acid metabolism and TIE

Altered metabolism of arachidonic acid, an unsaturated fatty acid, is also a characteristic of tumors, especially those associated with inflammation, such as colorectal cancer, lung cancer and bladder cancer.^84^ Most of these tumors show increased expression of prostaglandin E2 (PGE2) and cyclooxygenase-2 (COX-2), two key molecules of the arachidonic acid metabolic pathway. PGE2 and COX-2 overexpression in tumors may be one of the potential mechanisms underlying TIE. First, COX-2 induces DCs to secrete interleukin-10 (IL-10) and transforming growth factor-β (TGF-β), which in turn activate regulatory T cells (Tregs) and promote immunosuppression.^85^ Second, PGE2 and COX-2 induce the expression of the Treg-specific transcription factor forkhead box P3 (FOXP3), thus increasing the activity of Tregs.^86^ In addition, PGE2 directly inhibits lymphocyte function by increasing cellular cAMP levels.^87^ miRNAs have been found to regulate COX-2 and PGE2 in tumors (Table 2). For example, miR-206 inhibits PGE2-induced proliferation and metastasis of colon cancer cells by targeting the transmembrane 4 L six family member 1 (TM4SF1) protein,^88^ while miR-128,^89^ miR-146a,^90^ miR-101,^91^ and miR-143^92^ can inhibit tumor progression by decreasing COX-2. The involvement of ncRNAs other than miRNAs in the regulation of COX-2 and PGE2 has not yet been reported. Therefore, the idea that ncRNAs directly regulate the metabolism of arachidonic acid and affect immune escape may be a potential mechanism of TIE.

### Tryptophan metabolism and TIE

Indoleamine 2,3-dioxygenase (IDO) and tryptophan 2,3-dioxygenase (TDO) are the two key enzymes involved in tryptophan metabolism, and they can be manipulated by tumors to evade immune surveillance. IDO and TDO are highly expressed in tumor cells and can promote the recruitment of Tregs in the TME and induce immunosuppression via increased secretion of the immunosuppressive factors IL-6, IL-10, and TGF-β,^93–95^ which can help tumors achieve TIE. ncRNAs have been reported to regulate IDO and TDO expression in tumor cells (Table 2). High expression of lncRNA SNHG1 in breast cancer specifically inhibits miR-448, thereby increasing the expression of IDO, promoting Treg differentiation, enhancing immunosuppression, and promoting tumor proliferation and metastasis,^96^ also providing an advantage for the occurrence of TIE. lncRNA MALAT1 promotes immunosuppression by inducing IDO expression.^97^ In addition, studies confirmed that miR-153-3p specifically inhibits IDO expression and participates in the development of acute graft-versus-host disease in vitro and in vivo.^98^ All of the above findings are based on protein level regulation research. Whether ncRNAs can regulate tryptophan metabolism at the functional level and further affect TIE may also become a new potential target research mechanism.

## Cancer stem cell-like phenotype and TIE

**Fig. 3:**
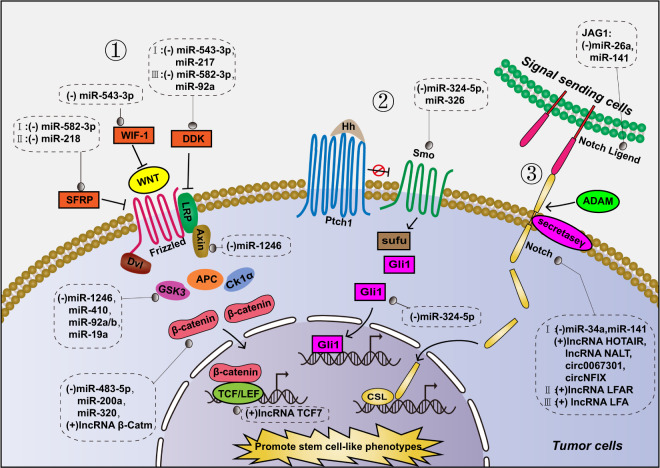
Regulation of TIE-associated stem cell signaling pathways by ncRNAs. The pathways include the following: 1. WNT signaling pathway; 2. Hedgehog signaling pathway; and 3. Notch signaling pathway

Acquiring a stem cell-like phenotype is considered another strategy adopted by tumors to achieve TIE. This is attributed to the low immunogenicity of cancer stem cells, and their immunoregulatory properties can inhibit the antitumor immune response and help them evade immune recognition.^99^ The WNT, Notch, and Hedgehog signaling pathways are closely associated with the development of stem cell-like phenotypes in most tumor cells. These three signaling pathways are abnormally activated in tumors to promote the transcription of target genes, thus improving the tumor stem cell-like phenotype (Fig. 3).^100^ ncRNAs were found to activate these signaling pathways by regulating key proteins involved in these pathways, thereby promoting stem cell-like phenotypes in tumor cells (Table 3, Fig. 3), which is also advantageous for the occurrence of TIE. miR-1246 activates the WNT pathway and promotes tumor stemness by inhibiting two components of the WNT pathway, Axin2 and glycogen synthase kinase 3β (GSK-3β), in liver cancer.^101^ In colorectal cancer and glioma, circRNA 100290 and circRNA 0000177 upregulate Frizzled receptor expression by acting as molecular sponges for miR-516b and miR-638, respectively, thereby activating the WNT pathway and promoting the stem-cell phenotype of tumor cells.^102,103^ miR-34a targets Notch1, an important membrane surface receptor of the Notch pathway, thereby inhibiting the stem cell-like phenotype of breast cancer cells.^104^ miR-324-5p has been found to significantly reduce the activation of the Hedgehog pathway by inhibiting Smoothened (Smo) and Gli zinc‐finger transcription factor-1 (Gli1), thereby inhibiting the stem-cell phenotype of multiple myeloma cells.^105^

**Table 3:** ncRNAs influence TIE via regulating tumor stem cell-like phenotype by targeting stem cell pathway or stemness genes

ncRNAs	Target genes	Function	Type of cancer	Refs.
*WNT signaling pathway*				
miR-543-3p	WIF-1	Activates WNT pathway by inhibiting WTF-1, and promotes stem cell-like phenotype of tumor cells	Bladder cancer	^372^
miR-218 and miR-1301-3p	SFRP	Activate WNT pathway by inhibiting SFPR (an inhibitor of WNT pathway), and promote stem cell-like phenotype of tumor cells	Triple negative breast cancer, Prostate cancer	^373,374^
miR-543-3p, miR-217, circRNA 0006427 and circRNA 0000523	DDK1	Activate/inhibit the WNT pathway by up-/downregulate the WNT pathway inhibitor DDK1, and regulate stem cell-like phenotype of tumor cells	Bladder cancer, Hepatocellular carcinoma, Lung adenocarcinoma, Colorectal cancer	^372,375–377^
circRNA CBFB, 100290, 0000177 and NEK6	Frizzed receptor3/4/7/8	Activate Notch pathway by upregulating Frizzed receptor4/7/8, and promote stem cell-like phenotype of tumor cells	Chronic Lymphocytic Leukemia, Colorectal cancer, Glioma, Thyroid cancer	^102,103,378,379^
miR-1246, 410, 92a and 19	GSK-3β	Inhibit the WNT pathway by inhibiting the expression of GSK-3β, and inhibit stem cell-like phenotype of tumor cells	Liver cancer, NSCLC, Colorectal cancer, Lung cancer	^101,380–382^
miR-1246	Axin	Inhibits the WNT pathway by inhibiting the expression of Axin, and inhibits stem cell-like phenotype of tumor cells	Liver cancer	^101^
circRNA 0002052 and circRNA 0009361	APC	Inhibit the WNT pathway by promoting the expression of APC, and inhibit stem cell-like phenotype of tumor cells	Osteosarcoma	^383,384^
miR-320, miR-200a and lncRNA β-Catm	β-catenin	Inhibit/activate the WNT pathway by up-/downregulate β-catenin, and regulate stem cell-like phenotype of tumor cells	Prostate cancer, Liver cancer	^385–387^
lncRNA TCF7	TCF7	Activates the WNT pathway by upregulating the transcription factor TCF7, and promotes stem cell-like phenotype of tumor cells	Colorectal cancer	^388^
*Hedgehog signaling pathway*				
miR-324-5p	Smo, Gli	Inhibits the Hedgehog pathway by downregulate Smo and Gli, and inhibits stem cell-like phenotype of tumor cells	Multiple myeloma	^105^
miR-326	Smo	Inhibits the Hedgehog pathway by inhibiting Smo, and inhibits stem cell-like phenotype of tumor cells	Chronic myeloid leukemia	^389^
*Notch signaling pathway*				
miR-26a and miR-141	JAG1(Notch ligand)	Inhibits the Notch pathway by inhibiting the expression of JAG1, and inhibits stem cell-like phenotype of tumor cells	Osteosarcoma, Glioblastoma	^390,391^
lncRNA HOTAIR, lncRNA NALT, circRNA NFIX, circRNA ASH2L	Notch1	Activates Notch pathway by upregulating the expression of Notch1, and promote stem cell-like phenotype of tumor cells	Intervertebral disc degeneration, Acute lymphoblastic leukemia, Glioma, Pancreatic ductal adenocarcinoma	^392–395^
miR-34a	Notch1	Inhibits Notch pathway by downregulating Notch1, and inhibits stem cell-like phenotype of tumor cells	Breast cancer	^396^
lncRNA LFAR	Notch2/3	Activates the Notch pathway by upregulate Notch2/3, and promote stem cell-like phenotype of tumor cells	Hepatic stellate cells	^397^
*Stemness related genes*				
miR-34a	SOX2, NANOG and OCT3/4	Downregulate SOX2, Nanog, and OCT3/4, thereby inhibiting stem cell-like phenotype of tumor cells	Head and neck squamous cell carcinoma	^108^
miR-208a	LIN28, SOX2	Upregulate LIN28, SOX2, thereby inhibiting stem cell-like phenotype of tumor cells	Breast cancer	^398^
let- 7, miR-125, 9 and 30	LIN28	Downregulate LIN28, thereby inhibiting stem cell-like phenotype of tumor cells	A2780, T47D, MCF7 and HeLa cancer cell lines	^399^
miR-21	OCT4	Downregulates OCT4, thereby inhibiting stem cell-like phenotype of tumor cells	Liver cancer	^400^
lncRNA DYNC2H1-4, SNHG20 and HOTTIP	LIN28, Nanog, SOX2 and OCT4	Upregulate LIN28, Nanog, SOX2 and OCT4, and promote stem cell-like phenotype of tumor cells	Pancreatic cancer, Oral squamous cell carcinoma, Pancreatic cancer	^401–403^
lncRNA FEZF1-AS1, lncRNA ITGB1 and piRNA-823	Nanog, OCT4 and SOX2	Upregulate Nanog, SOX2 and OCT4, and promote inhibiting stem cell-like phenotype of tumor cells	Breast cancer, NSCLC, Multiple myeloma	^110,404,405^
lncRNA H19	LIN28	Enhances upregulate LIN28, and promotes the stem-cell-like phenotype	Breast cancer	^109^

*SFPR* secreted frizzled related protein, *DDK1* dickkopf-1, *APC* adenomatous polyposis coli, *JAG1* Jagged1

Octamer transcription factor-3/4 (OCT3/4), SRY-box 2 (SOX2), Nanog and LIN28 are genes related to the tumor stem cell-like phenotype that have been proven to be related to TIE.^106,107^ ncRNAs can also promote the tumor stem cell-like phenotype by directly or indirectly regulating those genes (Table 3). For example, miR-34a targets and inhibits the expression of SOX2, Nanog, and OCT3/4, thereby inhibiting the stem cell-like phenotype of head and neck squamous carcinoma cells.^108^ lncRNA H19 acts as a molecular sponge for let-7 to upregulate LIN28 and promote the stem cell-like phenotype of breast cancer cells.^109^ In a study of multiple myeloma, granulocyte-MDCSs increased the expression of SOX2, OCT4, and Nanog in multiple myeloma stem cells by promoting the expression of piRNA-823, which controlled tumor stemness through DNMT3B activation, thereby promoting the tumor stemness phenotype.^110^ The above research results provide preliminary evidence that these ncRNAs promote the development of TIE by targeting tumor stem cell-like phenotype-related pathways and genes. By inhibiting this process, we may be able to improve resistance to immunotherapy.

## Epithelial–mesenchymal transformation (EMT) and TIE

EMT involves molecular changes that transform epithelial cells into mesenchymal cells, and such transformation enables the cells to lose cell-cell adhesion and apical-basal polarity. Therefore, EMT in tumor cells is essential to promote the metastasis of epithelial tumors.^111,112^ Several studies have reported that EMT may also induce immunosuppression and help tumors achieve TIE. Snail-induced EMT stimulates the production of immunosuppressive factors such as TGF-β and thrombospondin-1 (TSP-1), which could damage DCs, decrease the expression of costimulatory molecules, and increase the expression of IDO, thus indirectly inducing Treg differentiation and promoting immunosuppression.^113^ A study also proved that Snail-induced EMT in melanoma cells are resistant to CTL lysis.^113^ Furthermore, compared with breast cancer epithelial cells, mesenchymal cells generated via EMT in breast cancer cells show low expression of MHC class Ι molecules and high expression of programmed death ligand 1 (PD-L1), thereby inducing immune resistance and promoting TIE.^114^ Therefore, tumor cell EMT can promote immunosuppression in many ways and become one of the potential driving forces of TIE.

EMT is mainly mediated by three transcription factors: zinc-finger E-box-binding 1 (ZEB1), Snail, and Twist1.^115^ These transcription factors can decrease epithelial cadherin (E-cadherin) and increase neural cadherin (N-cadherin) and vimentin, thereby promoting the occurrence of EMT.^112^ There is also evidence that these transcription factors are associated with immune escape.^113,116^ miRNAs such as miR-21, miR-137, miR-34a, and miR-106a/b are known to regulate EMT by targeting these transcription factors (Table 4). In colorectal cancer, miR-21 can downregulate the expression of Snail and E-cadherin to inhibit EMT.^117^ In ovarian cancer, miR-137 and miR-34a can also downregulate Snail expression to inhibit EMT,^118^ while miR-106a can upregulate the expression of Snail and promote EMT in glioma cells.^119^ In hepatocellular carcinoma (HCC), miR-106a/b can inhibit EMT by downregulating Twist1.^120^ In contrast, miR-23a can upregulate Twist1 expression and promote EMT and cisplatin resistance in tongue squamous cell carcinoma.^121^ In addition to miRNAs, other ncRNAs have also been found to modulate EMT by targeting these transcription factors. In pancreatic cancer, lncRNA PVT1 promotes EMT by upregulating the expression of ZEB1, Snail, and N-cadherin and downregulating E-cadherin expression.^122^ In bladder cancer, lncRNA UCA1 can promote EMT by upregulating the expression of N-cadherin, vimentin, and Snail and downregulating the expression of E-cadherin; however, as a competitive endogenous RNA (ceRNA) of miR-145, it also upregulates the expression of ZEB1.^123^ In melanoma, circRNA 0084043 upregulates Snail expression by acting as a ceRNA of miR-153-3p, thus promoting EMT.^124^ These dysregulated ncRNAs accelerate EMT by regulating transcription factors in tumors, but their further impact on TIE remains unknown. Whether blocking the mechanism by which these ncRNAs regulate EMT can be conducive to inhibiting TIE and improving the effect of immunotherapy is worth further study.

**Table 4:** ncRNAs influence TIE via regulate EMT by targeting EMT- related transcription factors

Target genes	ncRNAs	Function	Type of cancer	Refs.
Twist1				
	miR-543, 300, 186, 137, 720, 580, 539, 33a, 33b, 520d-5p, 106b, 675,337-3p and 151-5p	Inhibit EMT of tumor cells by targeting and downregulating Twist1, an EMT related transcription factor	Endometrial cancer, Epithelial ovarian cancer, Gastrointestinal stromal tumor, Breast cancer, Osteosarcoma, Melanoma, endometrial carcinoma, HCC, Lung carcinoma	^120,406–417^
	miR-23a, lncRNA AK027294, lncRNA ROR and lncRNA AFAP1-AS1	Promote EMT of tumor cells by targeting and upregulating Twist1, an EMT related transcription factor	Tongue squamous cell carcinoma, Colorectal cancer, Gallbladder cancer	^121,418–420^
Snail				
	miR-21, 137, 34a, 491-5p, 22, 363, 30, 145, 153, 410-3p	Target and downregulate transcription factor Snail, and then inhibit EMT of tumor cells	Colorectal cancer, Ovarian cancer, Gastric cancer, Bladder cancer, Lung cancer, Osteosarcoma, HCC, Breast cancer	^117,118,421–427^
	miR-106a, circRNA 0084043, circRNA PRMT5, lncRNA PVT1 and lncRNA UCA1	Target and upregulate transcription factor Snail, and then promote EMT of tumor cells	Glioma, Melanoma, Bladder carcinoma, Pancreatic cancer, Breast cancer	^119,122,124,428,429^
ZEB1				
	miR-203, 873, 205-5p, 5702, 126, 186-5p	Target and downregulate transcription factor ZEB1, and then inhibit EMT of tumor cells	Gastric cancer, Breast cancer, Prostate cancer, NSCLC, Cervical cancer, Colorectal cancer	^430–435^
	lncRNA MALAT1, ZEB1-AS1, SNHG16, NNT-AS1, HOTTIP, NEAT1, ZNF469-3, TP73-AS1, circRNA TSPAN4 and circRNA PVT1	Target and upregulate transcription factor ZEB1, and then promote EMT of tumor cells	HCC, NSCLC, Osteosarcoma, Breast cancer, Glioma, Nasopharyngeal carcinoma, Lung adenocarcinoma, Gastric cancer	^436–445^

## Immunosuppressive cells and TIE

### Tregs

Tregs, cells that act as immunosuppressive agents in the body, play important roles in TIE. First, Tregs produce immunosuppressive factors such as IL-10, IL-35, and TGF-β, which inhibit the function of antitumor T cells.^125^ Second, Tregs inhibit T cell function by expressing coinhibitory factors such as cytotoxic T lymphocyte antigen 4 (CTLA-4), programmed cell death protein 1 (PD-1), and PD-L1.^126^ In addition to directly affecting T cells, Tregs can also inhibit T cell activation by targeting the maturation and activity of DCs.^125^ miRNAs play an essential role in Treg maintenance and function. Foxp3-dependent regulation of miR-155 contributes to the proliferative activity and competitive fitness of Tregs.^127^ miR-146a, highly expressed in Tregs, was found to be able to regulate Treg function, and loss of miR-146a led to increased production of the proinflammatory Th1 cytokine IFN-γ by Foxp3^+^ Tregs, and transferring purified miR-146a-deficient Tregs together with Foxp3 KO CD4^+^ effector T cells into lymphopenic recipients failed to repress Th1 responses.^128^ In tumors, abnormally expressed ncRNAs have been found to regulate Tregs. The miR-17-92 cluster is expressed in many human blood tumors, and studies have shown that the miR-17-92 cluster can regulate the number of Tregs by targeting Bim.^129^ Some lncRNAs are also involved in Treg regulation. lncRNA HULC, which is highly expressed in HCC, downregulates p18 in liver cirrhosis to affect Treg differentiation.^130^ In gastric cancer, lncRNA POU3F3 promotes the distribution of Tregs among surrounding T cells by recruiting TGF-β and activating the TGF-β pathway.^131^ lncRNA SNHG1 regulates Treg differentiation by targeting miR-448/IDO in breast cancer.^96^

### Myeloid-derived suppressor cells (MDSCs)

As a group of heterogeneous cells derived from the bone marrow, MDSCs are precursors of DCs, macrophages, and/or granulocytes. MDSCs can significantly inhibit the cellular immune response and are one of the important driving forces of TIE. First, MDSCs induced by HMGB1 and those with myeloid differentiation potential can mediate TIE by producing high levels of IL-10, inhibiting the activation of antigen-driven CD4^+^ and CD8^+^ T cells and the expression of L-selectin in circulating naive T cells.^132^ Second, tumor cells can also inhibit the function of T cells, NK cells, and DCs by altering the expression of cellular stress sensor C/EBP homologous protein (Chop) and the secretion of IL-6 by MDSCs.^133^ Moreover, the proliferation of CXCR2^+^CD11b^+^Ly6G^hi^ MDSCs induced by CXCR2 ligands produced by tumor cells inhibits T cell proliferation by L-arginine depletion and exerts local immunosuppressive effects.^134^ Furthermore, MDSCs can inhibit the host antitumor immune response by inducing Tregs.^135^ MDSC-derived NO reacts with superoxide to produce peroxynitrite (PNT), which directly inhibits T cells by nitrating the T cell receptors (TCRs) present on the surface of tumor-specific T cells and reducing the reactivity of the associated antigen-MHC complexes.^136^ ncRNAs have been found to be involved in regulating the immunosuppressive activity of MDSCs. In tumor-bearing mice, *lncRNA PVT1* regulates the immunosuppressive activity of MDSCs, and *lncRNA PVT1* knockdown significantly inhibits the immunosuppressive activity of MDSCs.^137^ Similarly, lncRNA HOTAIRM1 negatively regulates the immunosuppressive activity of MDSCs by targeting HOXA1 in lung cancer.^138^ In addition, some ncRNAs regulate the proliferation, differentiation, and recruitment of MDSCs. miR-34a promotes the proliferation of MDSCs by inhibiting their apoptosis,^139^ whereas miR-9 regulates the differentiation and function of MDSCs by targeting runt-related transcription factor 1 (Runx1).^140^ Conversely, the lack of miR-155 in B16-F10 melanoma and Lewis lung carcinoma cell lines leads to the recruitment of MDSCs in the TME and subsequently enhances immunosuppression.^141^

### Tumor-associated macrophages (TAMs)

TAMs have been proven to be the most significant immune cells in the tumor stroma, accounting for more than 50% of the total number of immune cells in the tumor stroma, which are divided into two groups, M1 and M2. M1 macrophages play an important role in the innate immune response to pathogen invasion, whereas M2 macrophages are alternately activated by IL-4, IL-10, IL-13, and glucocorticoids. Studies have found that macrophages in tumor tissues mostly have the phenotype and function of M2 macrophages, which is one of the important driving forces for TIE. First, M2 macrophages produce high levels of the immunosuppressive factor IL-10 in breast tumors, which can inhibit IL-12 expression in tumor DCs and subsequent IL-12-mediated CTL activation, thereby blocking the CTL-dependent antitumor immune response.^142^ Second, M2 macrophages directly inhibit the activity of CD8^+^ T cells via the expression of coinhibitory factors such as PD-L1 and B7-H4 but indirectly play an antitumor role by promoting the recruitment of CCL22-mediated Tregs.^143^ Tumor cells can secrete certain cytokines (CCL2, CSF1, and CXCL12) to promote M2 macrophage recruitment in the TME, thus inducing immunosuppression. In the mouse sarcoma MS-K cell line, *miR-342* inhibits the expression of CXCL12 and reduces the recruitment and activation of M2 macrophages.^144^ lncRNA NIFK-AS1 inhibits the M2 polarization of macrophages by targeting miR-146a, thus reducing immunosuppression and inhibiting the proliferation, migration, and invasion of endometrial cancer.^145^

In conclusion, immunosuppressive Tregs, MDSCs and M2 macrophages can achieve immunosuppression in many ways, thus reducing the recognition and killing of tumors by the immune system. However, these functions are utilized by ncRNAs to help tumors achieve TIE, which may be effective targets to prevent TIE and improve tumor immunotherapy.

## Immune checkpoint molecules and TIE

**Fig. 4:**
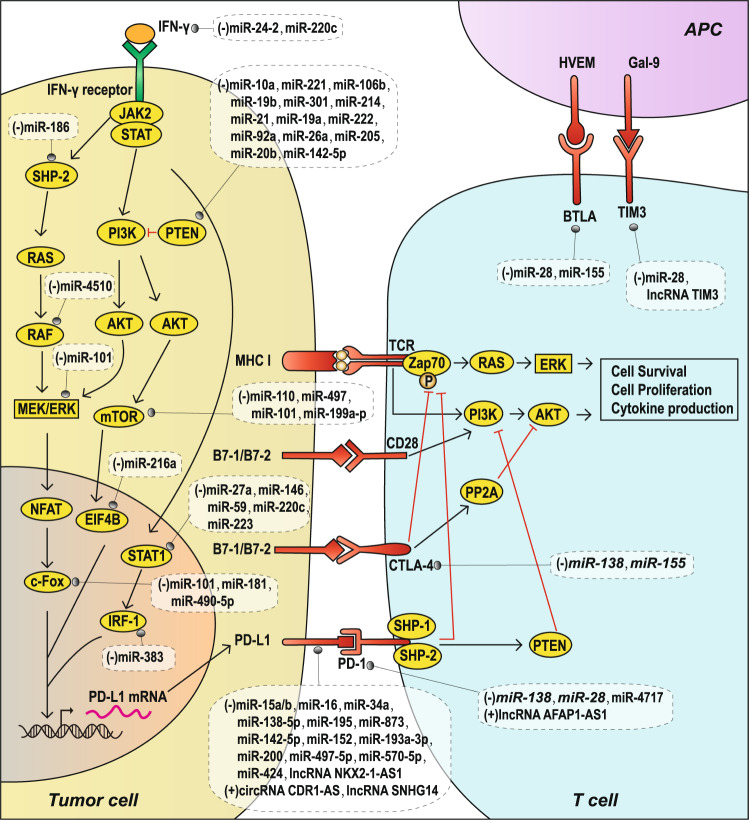
TIE and immune checkpoint molecules regulated by ncRNAs. PD-L1 expression induced by the IFN-γ signaling pathway and PTEN/PI3K/AKT/mTOR pathway, as well as the regulation of these pathways by ncRNAs (**left**).T-cell-activated receptors/ligands (TCR/MHC-I and CD28/B7-1/2) and immune checkpoint molecular receptor-ligands (PD-1/PD-L1, CTLA-4/B7-1/2, BTLA/HVEM and TIM3/Gal-9) regulated by ncRNAs (**middle and upper right**). The red “T” symbol represents inhibitory modification

Immune checkpoints refer to a plethora of inhibitory pathways hardwired into the immune system that are crucial for maintaining self-tolerance and modulating the duration and amplitude of physiological immune responses in peripheral tissues to minimize collateral tissue damage. It is now clear that tumors coopt certain immune checkpoint pathways as a major mechanism of tumoral immune resistance, particularly against T cells that are specific for tumor antigens.^146^ The expression of immune checkpoint proteins can be dysregulated in tumors as an important TIE mechanism.^147^ Therefore, the study of immune checkpoints is particularly essential to prevent TIE. The currently known TIE-related immune checkpoint molecules are CTLA-4, PD-1, PD-L1, T-cell immunoglobulin and mucin domain-containing protein 3 (TIM-3), and B and T lymphocyte attenuator (BTLA) (Fig. 4). Next, we will summarize the research progress on ncRNAs in the regulation of immune checkpoints (Table 5).

**Table 5:** ncRNAs influence TIE via regulating immune checkpoint molecules

Target genes	ncRNAs	Function	Type of cancer	Refs.
CTLA-4				
	*miR-138* and *miR-155*	Downregulate CTLA-4 on tumor-infiltrating T cells directly, promote the recognition and killing of tumor cells by the immune system	Mouse gliomas, Mouse melanoma	^151,152^
PD-L1				
	miR-424, 16, 195, 34a, 15a, 15b, 16, 193a-3p, 873, 497-5p, 570, 152, 142-5p, 138-5p and lncRNA NKX2-1-AS1	Downregulate PD-L1 on tumor cells directly, inhibit immunosuppression and preventing TIE	Ovarian cancer, Prostate cancer, AML, Malignant pleural mesothelioma, Breast cancer, Clear cell renal cell carcinoma, Gastric cancer, Pancreatic cancer, Colorectal cancer, Lung carcinoma	^159–162,164,174,446–450^
	circRNA CDR1-AS	Upregulates PD-L1 on tumor cells directly, promotes immunosuppression and TIE	Colon cancer	^173^
	lncRNA SNHG14	Upregulates PD-L1 by SNHG14/miR-5590-3p/ZEB1 positive feedback loop, promotes immunosuppression and TIE	Large B cell lymphoma	^172^
	miR-24-2 and miR-200c	Downregulate PD-L1 by inhibiting the IFN-γ in IFN-γ signaling pathway, inhibit immunosuppression and TIE	Cervical cancer	^165^
	miR-186	Downregulates PD-L1 by inhibiting SHP-2 in IFN-γ signaling pathway, inhibits immunosuppression and TIE	Oral squamous cell carcinoma	^166^
	miR-4510	Downregulates PD-L1 by inhibiting RAF1 in IFN-γ signaling pathway, inhibits immunosuppression and TIE	HCC	^451^
	miR-101	Downregulates PD-L1 by inhibiting MEK1 in IFN-γ signaling pathway, inhibits immunosuppression and TIE	Nasopharyngeal carcinoma	^452^
	miR-27a, 145, 150, 223 and 200c	Downregulate PD-L1 by inhibiting STAT1 in the IFN-γ signaling pathway, inhibit immunosuppression and TIE	Cervical cancer, Colon cancer, Adult T cell leukemia/lymphoma, Cervical cancer	^165,167,453^
	miR-383	Downregulates PD-L1 by inhibiting IRF1 in the IFN-γ signaling pathway, inhibit immunosuppression and TIE	Testicular embryonal carcinoma	^454^
	miR-101, 181b and 490-5p	Downregulate PD-L1 by inhibiting c-FOS in the IFN-γ signaling pathway, inhibit immunosuppression and TIE	Osteosarcoma, Glioma, Bladder cancer	^455–457^
	miR-10a, 19a, 19b, 106b, 221, 222, 20b, 21, 130b, 92a, 26a, 205, 214, 301a and 142-5p	Upregulate PD-L1 by inhibiting PTEN in PTEN/PI3K/AKT/mTOR pathway, promote immunosuppression and TIE	NSCLC, Gastric cancer, Colorectal cancer, Lung cancer, Nasopharyngeal carcinoma, Ovarian cancer, Breast cancer, NSCLC	^168,169,458–466^
	miR-100, 101, 199a-3p and 497	Upregulate PD-L1 by downregulating mTOR in PTEN/PI3K/AKT/mTOR pathway, promote immunosuppression and TIE	Bladder cancer, Osteosarcoma cell, Endometrial cancer cell, Ovarian cancer	^170,467–469^
	miR-216a	Upregulates PD-L1 by downregulating EIF4B in PTEN/PI3K/AKT/mTOR pathway, promotes immunosuppression and TIE	NSCLC	^171^
PD-1				
	*miR-28, 138* and 4717	Downregulate PD-1 directly on tumor-infiltrating T cells, promotes the activity and function of T cells and inhibit TIE	Mouse melanoma, Mouse glioma, Chronic HBV	^151,179,470^
	ncRNA AFAP1-AS1	Up- regulates PD-1 expression on tumor-infiltrating lymphocytes, inhibits the activity and function of lymphocytes and promotes TIE	Nasopharyngeal carcinoma	^181^
TIM-3				
	miR-28	Reduces T cell exhaustion and increasing TNF-α and IL-2 secretion by downregulating TIM-3 directly, thereby inhibiting TIE	Melanoma	^179^
	lncRNA Tim3	Exacerbates CD8^+^ T cell exhaustion by specifically binding to TIM-3, thereby promoting TIE	HCC	^184^
BTLA				
	miR-28 and miR-155	Downregulate BTLA directly and enhance antitumor immune response and inhibit TIE	Melanoma, CD4+ T cell	^179,186^

### CTLA-4

Activated T cells play vital roles in antitumor immunity, and T cell activation depends on two signals: one involving an interaction between TCR and MHC molecules and another involving the costimulatory signal between CD28 and B7-1/2. To ensure a balance in the function of the immune system, CTLA-4, a homolog of CD28, binds to B7-1/2 and forms an inhibitory signal for T lymphocytes with activated TCRs.^148^ CTLA-4 binds to B7-1/2 with an affinity higher than that of CD28 and inhibits AKT phosphorylation by activating phosphatase protein phosphatase 2A (PP2A), thereby inhibiting subsequent T cell activation and function (Fig. 4).^149^ In addition, CTLA-4 inhibits the formation of ζ chain-associated protein kinase 70 (Zap70), thereby affecting TCR signaling and ultimately inhibiting T cell function and promoting T cell apoptosis (Fig. 4).^150^ Therefore, overexpression of CTLA-4 on the surface of tumor-infiltrating T cells will enhance immune suppression by inhibiting the activation and function of T cells and promoting T cell apoptosis, thus helping tumor cells achieve TIE. Some miRNAs with abnormal expression in tumor cells can regulate the surface expression of CTLA-4 in tumor-infiltrating T cells, thereby inhibiting the antitumor immune response mediated by T cells and promoting TIE (Table 5, Fig. 4). In mice with glioma, *miR-138* significantly reduces the expression of CTLA-4 and PD-1 on the surface of tumor-infiltrating T cells.^151^ In mice with melanoma*, miR-155* inhibits CTLA-4 expression on the surface of tumor-infiltrating T cells and promotes the host antitumor immune response.^152^

### PD-L1

PD-L1 (B7-H1) is a transmembrane protein expressed by T cells, B cells, and various tumor cells.^153^ Binding of PD-L1 with PD-1 on the surface of CTLs can inhibit the proliferation of CTLs and suppress the secretion of CTL cytokines such as IL-2, thereby affecting the function of CTLs and promoting TIE.^146^ The overexpression of PD-L1 on the surface of tumor cells through different mechanisms can enhance immunosuppression and promote immune escape. Currently, PD-L1 is the main target for tumor immunotherapy, and some PD-L1 monoclonal antibodies, such as durvalumab and atezolizumab, have shown good clinical therapeutic effects.^154,155^ In addition to factors that directly regulate PD-L1 mRNA, studies have shown that the transcription and expression of PD-L1 in tumor cells are strongly dependent on the interferon-γ (IFN–γ) signaling pathway (Fig. 4).^156,157^ In addition, PD-L1 is also regulated by the phosphatase and tensin homolog deleted on chromosome ten (PTEN)/phosphatidylinositide 3-kinase (PI3K)/AKT/mammalian target of rapamycin (mTOR) signaling pathway in tumor cells (Fig. 4).^158^ The absence of PTEN promotes AKT and mTOR phosphorylation, which in turn leads to increased PD-L1 translation (Fig. 4).^158^ miRNAs can affect PD-L1 expression in tumor cells by directly targeting the PD-L1 mRNA or targeting the intermediate links of related signaling pathways (Table 5, Fig. 4). miRNAs such as miR-34a,^159^ miR-138-5p,^160^ miR-142-5p,^161^ miR-152,^162^ miR-200,^163^ and miR-424^164^ directly inhibit PD-L1 by targeting PD-L1 mRNA in different tumor cells. In addition, miRNAs such as miR-24-2,^165^ miR-186,^166^ miR-27a,^165^ and miR-145^167^ alter PD-L1 expression by regulating the IFN-γ signaling-mediated transcription and expression of PD-L1. Other miRNAs, such as miR-10a,^168^ miR-221,^169^ miR-100,^170^ and miR-216a,^171^ alter PD-L1 expression by targeting the PTEN/PI3K/AKT/mTOR signaling pathway. In addition to miRNAs, a number of lncRNAs and circRNAs are also involved in regulating the expression of PD-L1 (Table 5, Fig. 4). lncRNA SNHG14 activates PD-L1 expression at the transcriptional level via ZEB1 and miR-5590-3p, thus promoting immune escape of diffuse large B cell lymphoma.^172^ circRNA CDR1-AS positively regulates PD-L1 levels and leads to poor prognosis in patients with colorectal cancer.^173^ lncRNA NKX2-1-AS1 inhibits PD-L1 expression in tumor cells at the transcriptional level, thus inhibiting immunosuppression and preventing TIE in lung carcinoma cells.^174^

### PD-1

PD-1 is mainly expressed on the surface of activated T cells, B cells, and DCs.^175^ The interaction between PD-L1 and PD-1 inhibits TCR-mediated T cell activation (Fig. 4) and is a significant mechanism of TIE. Mechanistically, when PD-L1 and PD-1 interact, intracellular tyrosine of the ligand-bound PD-1 is phosphorylated and thus activated. Src homology 2-containing protein tyrosine phosphatase 1 (SHP-1) and SHP-2 are then recruited to the C-terminal immunoreceptor tyrosine-based switch motif (ITSM) of PD-1, and they inhibit the activation of the RAS/extracellular signal-regulated kinase (ERK) signaling pathway via ZAP70 dephosphorylation (TCR activation signals). This promotes T cell apoptosis and inhibits the proliferation of T cells and secretion of cytokines such as IL-2 (Fig. 4).^176,177^ In addition, PD-1 can also activate PTEN, indirectly inhibiting the TCR-mediated PI3K/AKT signaling pathway (Fig. 4).^178^ Multiple miRNAs and lncRNAs have been found to regulate PD-1 expression on the surface of tumor-infiltrating T cells and promote immunosuppression, thus promoting TIE (Table 5, Fig. 4). In melanoma-bearing mice, *miR-28* was found to specifically inhibit PD-1 expression on tumor-infiltrating T cells and prevent T cell exhaustion, which can enhance the antitumor immune response, whereas transfection with *miR-28* inhibitors increased the number of PD-1-positive exhausted T cells.^179^*miR-138* inhibits the expression of PD-1 and CTLA-4 on tumor-infiltrating T cells, promotes the activity and function of T cells, and inhibits tumor development in mouse GL261 glioma cells.^151^ miR-4717 was found to inhibit PD-1 expression in the lymphocytes of patients with chronic HBV infection, the leading cause of HCC.^180^ In addition, lncRNA AFAP1-AS1 positively regulates PD-1 expression on tumor-infiltrating lymphocytes in nasopharyngeal carcinoma.^181^

### TIM-3

TIM-3 is an immune checkpoint molecule expressed on the surface of DCs, NK cells, Tregs, macrophages, and IFN-γ-producing T cells.^182^ TIM-3 inhibits the function of type 1T helper cells and the secretion of several immune factors, such as IFN-γ and TNF.^182^ In addition, as an immune checkpoint molecule, TIM-3 can also inhibit antitumor immunity by depleting tumor-infiltrating T cells.^183^ Therefore, in tumors, regulating the expression of TIM-3 is also one of the mechanisms to achieve TIE, and ncRNAs are significant players in this regulation. For example, in HCC, lncRNA Tim3 can stimulate CD8^+^ T cell exhaustion and promote the survival of these exhausted CD8^+^ T cells by specifically binding to TIM-3, thereby inhibiting the T cell-mediated antitumor immune response and promoting TIE.^184^ In melanoma, miR-28 inhibits the expression of TIM-3, PD-1, and CTLA-4, thereby reducing T cell exhaustion and increasing TNF-α and IL-2 secretion, which could enhance the antitumor immune response and inhibit TIE (Table 5).^179^

### BTLA

Similar to PD-1 and CTLA-4, BTLA is also an inhibitory receptor on the surface of T cells, which could be used by tumors to achieve immune escape. It is expressed on both type 1 and type 2 T helper cells but is not expressed on highly polarized type 2 T helper cells.^185^ BTLA reduces IL-12 secretion by cross-linking with the antigen receptor, inducing its phosphorylation, and binding to the tyrosine phosphatase SHP-1/2 containing the Src homology 2 (SH2) domain.^185^ Moreover, BTLA-deficient T cells show increased proliferation.^185^ As mentioned in the previous section, in melanoma, miR-28 can inhibit the expression of BTLA, PD-1, and CTLA-4 in T cells, which could enhance the antitumor immune response and inhibit TIE.^179^ In addition, miR-155 can also inhibit BTLA expression, thereby weakening the inhibitory effect of BTLA on T cell activation (Table 5).^186^

In summary, regulating the expression of immune checkpoints on the surface of tumor cells or immune cells is a significant strategy for tumors to achieve immune escape, and ncRNAs play essential roles in this process. It may be an important strategy to inhibit TIE and improve the efficiency of immunotherapy by inhibiting the expression of these ncRNAs. At present, anti-immune checkpoint molecular targeting drugs have been used in the clinic, and good results have been achieved.^154,155^ However, targeted therapy for these ncRNAs has not been reported and needs further study.

## Cytokines in tme and TIE

### TGF-β

**Fig. 5:**
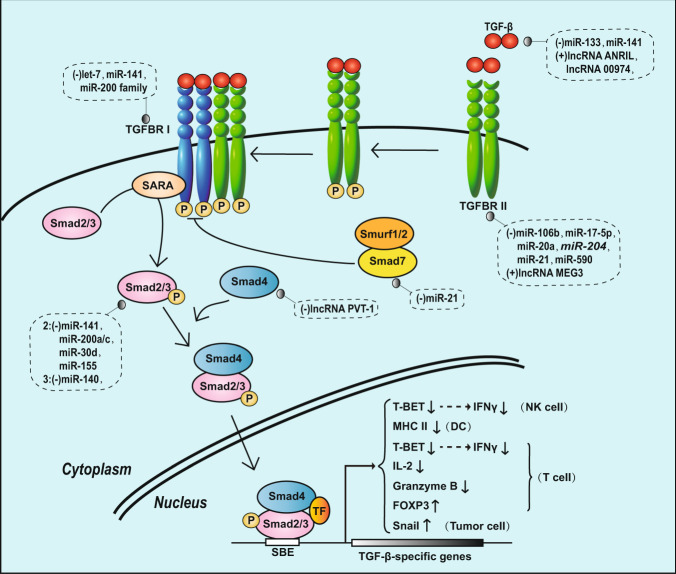
TIE and the canonical TGF-β signaling pathway regulated by ncRNAs

The role of TGF-β in tumorigenesis and development is contradictory. The TGF-β signaling pathway can inhibit tumor growth because the downstream signals of TGF-β family receptors can regulate the expression of DAPK, GADD45β, BIM, SHIP and other apoptosis genes, which can induce apoptosis of tumor cells.^187^ However, some studies have indicated that TGF-β signaling pathway activation can promote tumor growth and invasion and is critical for TIE.^188^ The mechanism is as follows: the TGF-β signaling pathway can induce the transcription of relevant target genes to inhibit the activation and/or functions of NK cells, DCs, and T cells and induce the differentiation of Tregs (Fig. 5).^187^ In NK cells, TGF-β signaling inhibits the expression of the transcription factor TBET, which inhibits IFN-γ expression, thereby inhibiting the function of NK cells.^189,190^ In DCs, TGF-β signaling inhibits MHC class II gene expression, thus inhibiting its antigen-presenting function.^191^ In T cells, TGF-β signaling silences the expression of TBET, thereby inhibiting the production of INF-γ.^192^ TGF-β signaling also inhibits the expression of IL-2 and granzyme B,^193,194^ thereby preventing the antitumor immune function of T cells. TGF-β signaling can also induce Treg differentiation by inducing the expression of FOXP3, the main transcription factor essential for the Treg phenotype, and then increase immunosuppression.^195^ Moreover, the TGF-β signaling pathway can also induce the expression of the transcription factor Snail in tumor cells and promote EMT, which may be another mechanism of TGF-β-induced immune escape (Fig. 5).^196^ Recent research has demonstrated that TGF-β promotes TIE via a mechanism independent of the canonical TGF-β signaling pathway. Tumor progression results in the downregulation of TGFBRII in T cells, enabling TGF-β to directly enter T cells. Once inside the cell, TGF-β molecules bind the Smad protein in mitochondria and disrupt the ATP-coupled respiration of T cells, thereby inhibiting their function and promoting TIE.^197^

TGF-β receptors are serine/threonine kinase receptors divided into type I and type II (TGFBR Ι and TGFBR II) receptors. Binding of the ligand TGF-β to TGFBR II induces the assembly of TGFBRΙ and TGFBR II complexes, and TGFBR II phosphorylates and activates TGFBR I, which then phosphorylates the two C-terminal serine residues of smad2 and Smad3. Phosphorylated Smad2 and Smad3 form heterotrimeric complexes with Smad4; this activated Smad complex enters the nucleus, interacts with other transcription factors, and regulates the expression of the corresponding target genes. Smad7 can form a complex with Smurf1/2 to inhibit the activity of TGFBR I, thereby inhibiting the activity of the pathway (Fig. 5).

ncRNAs in tumors can target components of the TGF-β signaling pathway or directly regulate TGF-β target gene transcription in different tumors, thus inhibiting the antitumor immune response and promoting TIE (Table 6, Fig. 5). For example, miR-133,^198^ miR-141,^199^ lncRNA ANRIL,^200^ and lncRNA 00974^201^ regulate the expression of TGF-β; Let-7,^202^ miR-141, and the miR-200 family^203^ target and regulate TGFBR Ι; *miR-106b*,^204^ miR-17-5p,^205^ miR-204,^206^ miR-20a,^207^ miR-21,^208^ miR-590^209^ and lncRNA MEG3^210^ target and regulate TGFBR II; miR-141, miR-200a/c, miR-30d^203^ and miR-155^211^ regulate Smad2; miR-140^212^ and lncRNA PVT1^213^ regulate the expression of Smad3 and Smad4, respectively; and miR-21 regulates the expression of Smad7 in cervical cancer.^214^ The abnormal expression of these ncRNAs in tumors can activate the TGF-β signaling pathway in different stages and then help tumors achieve immune escape by inhibiting a variety of immune cells, inducing the differentiation of immune cells and promoting EMT. According to this characteristic of tumors, blocking the activation of the TGF-β signaling pathway by targeting these ncRNAs may become a new direction for tumor immunotherapy.

**Table 6:** ncRNAs influence TIE via regulating cytokines

Target genes	ncRNAs	Function	Type of cancer	Refs.
TGF-β	miR-133 and miR-141	Downregulate TGF-β expression, inhibit immunosuppression and TIE, suppress tumor growth and invasion	Gastric cancer, Myocardial fibrosis	^198,199^
	lncRNA ANRIL and lncRNA 00974	Upregulate TGF-β expression, induce immunosuppression and TIE, promote tumor growth and invasion	Esophageal squamous cell carcinoma, HCC	^200,201^
TGFBR Ι	Let-7, miR-141 and miR-200 family	Downregulate the expression of TGFBRΙ, weaken TGF-β signal pathway, inhibit immunosuppression and TIE, suppress tumor growth and invasion	Thyroid carcinomas	^202,203^
TGFBR II	*miR-106b*, 17-5p, 204, 20a, 21, 590	Downregulate the expression of TGFBR II, weaken TGF-β signal pathway, inhibit immunosuppression and TIE, suppress tumor growth and invasion	Alzheimer’s disease, metastatic cancer, Lung cancer, Leiomyoma	^205–209,471^
	lncRNA MEG3	Upregulates the expression of TGFBR II, strengthens TGF-β signal pathway, induce immunosuppression and TIE, promotes tumor growth and invasion	Chondroma	^210^
Smad2	miR-141, 200a/c, 30d and 155	Downregulate the expression of Smad2, weaken TGF-β signal pathway, inhibit immunosuppression and TIE, suppress tumor growth and invasion	Thyroid carcinomas	^203,211^
Smad3	miR-140	Downregulates the expression of Smad3, weakens TGF-β signal pathway, inhibit immunosuppression and TIE, suppresses tumor growth and invasion		^212^
Smad4	lncRNA PVT1	Downregulates the expression of Smad4, weakens TGF-β signal pathway, inhibit immunosuppression and TIE, suppresses tumor growth and invasion	Colorectal cancer	^213^
Smad7	miR-21	Downregulates the expression of Smad4, strengthens TGF-β signal pathway, promotes immunosuppression and TIE	Cervical cancer	^214^
IL-6	miR-33a, 218, 125a, 34a, 217, 26a, 98, and 9	Downregulate IL-6, inhibit immunosuppression and TIE, suppress tumor growth and invasion	Gallbladder cancer, Lung cancer, Breast cancer, Cardiac myxoma, HCC, Melanoma, HeLa cell line	^220–227^
	lncRNA HOTTIP, 00460 and UICC	Directly upregulate IL-6, induce immunosuppression and TIE, promote tumor growth and invasion	Ovarian cancer, Nasopharyngeal carcinoma, Cervical cancer	^228–230^
STAT3	miR-551b-3p,	Upregulates STAT3 expression directly, induces immunosuppression and TIE, promotes tumor growth and invasion	Ovarian cancer	^231^
	miR-221, 222 and 18a	Downregulate STAT3 expression by negatively regulating the PDLIM2 or PIAS3, inhibit immunosuppression and TIE, suppress tumor growth and invasion	Gastric adenocarcinoma, Colorectal cancer	^232,233^
	lncRNA 00518, AB073614 and HOST2	Activate the JAK2/STAT3 signaling pathway, induce immunosuppression and TIE, promote tumor growth and invasion	Cervical cancer, Colorectal cancer, HCC	^235–237^
IL-10	miR-98	Downregulates IL-10, inhibits immunosuppression and TIE, suppresses tumor growth and invasion	HCC	^247^
	miR-194, miR-193b and lncRNA CCAT1	Upregulate IL-10, induce immunosuppression and TIE, promote tumor growth and invasion	Laryngeal cancer, Lymphoma, prostate cancer	^248–250^
VEGF	miR-638, 503, 497, 203, 200, 195, 190, 126, 93, 29b and 20	Downregulate VEGF directly, inhibit immunosuppression and TIE, suppresse tumor growth and invasion	HCC, Cervical cancer, Lung cancer, Oral cancer, Breast cancer	^254–263,265,266^
	miR-22, 107, 519c, 26a and 145	Downregulate VEGF indirectly, inhibit immunosuppression and TIE, suppress tumor growth and invasion	Colorectal cancer, HCC	^78,269–272^
	lncRNA TDRG1 and lncRNA HOTAIR	Upregulate VEGF directly, induce immunosuppression and TIE, promote tumor growth and invasion	Endometrial carcinoma, Nasopharyngeal carcinoma	^267,268^
	lncRNA H19 and lncRNA GAS5	Upregulate VEGF indirectly, induce immunosuppression and TIE, promote tumor growth and invasion	Mesenchymal stem cells, Colorectal cancer	^273,274^

*PDLIM2* PDZ and LIM domain protein 2, *PIAS3* Protein inhibitor of activated signal transducer and activator of transcription

### IL‐6

IL-6, commonly secreted by macrophages, DCs, MDSCs, and tumor cells, is a pleiotropic proinflammatory cytokine that is involved in almost all aspects of the immune system, from the infiltration of neutrophils at the site of infection to the generation of T cell responses. IL-6 can be rapidly induced and expressed in large quantities under the conditions of infection and autoimmunity and plays a key role in host defense by stimulating various cell populations (including promoting cytotoxic T cell differentiation, T cell population expansion and activation, and B cell differentiation).^215^ However, in addition to immune-stimulating effects, IL-6 can also lead to immunosuppression and TIE, most of which are mediated through the IL-6/JAK2/STAT3 signaling pathway. For example, the IL-6/JAK2/STAT3 signaling pathway can make the TME tend towards immunosuppress by attracting and activating MDSCs, TANs, and Tregs.^216^ In addition, the IL-6/JAK2/STAT3 signaling pathway has also been found to promote the tumor stem cell-like phenotype and EMT.^217–219^ In tumors, abnormal expression of ncRNAs can promote TIE by regulating the expression of IL-6 or the molecules involved in the IL-6/JAK2/STAT3 signaling pathway (Table 6). miR-33a,^220^ miR-218,^221^ miR-125a,^222^ miR-34a,^223^ miR-217,^224^ miR-26a,^225^ miR-98,^226^ miR-9,^227^ lncRNA HOTTIP,^228^ lncRNA 00460,^229^ and lncRNA UICC^230^ directly regulate IL-6 in different cancers. miR-551b-3p directly downregulates STAT3 expression in ovarian cancer cells,^231^ whereas miR-18a inhibits STAT3 by negatively regulating the expression of the E3 SUMO protein ligase PIAS3 in gastric adenocarcinogenesis.^232^ miR-221 and miR-222 inhibit STAT3 expression by targeting PDZ and LIM domain protein 2 (PDLIM2) in colorectal cancer.^233^ Moreover, miR-30 promotes the activation of the JAK/STAT3 pathway by inhibiting the expression of suppressor of cytokine signaling 3 (SOCS3) in glioma stem cells.^234^ lncRNA 00518, lncRNA AB073614, and lncRNA HOST2 activate the JAK2/STAT3 signaling pathway in cervical cancer, colorectal cancer, and HCC, respectively.^235–237^ These ncRNAs enhance the immunosuppression of the TME by regulating IL-6 expression and activating the IL-6/JAK2/STAT3 pathway, thus helping tumors achieve immune escape, which may be another target to block TIE and improve the effect of immunotherapy.

### IL-10

IL-10 is an immune cytokine produced by immune cells that plays a dual role in tumorigenesis and development.^238^ In the early tumor stage, the main role of IL-10 is to activate the immune system to kill tumor cells by stimulating NK cell- and CTL cell-mediated antitumor responses. However, with tumor development, some tumors (melanoma,^239^ lung cancer^240^ and bladder cancer^241^) have been found to be able to utilize the immunosuppressive effects of IL-10 to achieve TIE.^242^ Specific mechanisms are described below. First, IL-10 can inhibit the expression of MHC class II molecules on antigen-presenting cells and MHC class I molecules in tumor cells to inhibit tumor antigen presentation.^243,244^ Second, IL-10 can inhibit the activation of the CD28 costimulatory pathway in T cells, resulting in T cell dysfunction.^245^ Finally, tumor cells express IL-10 and IL-10 receptors by themselves, activate the downstream STAT3/Twist pathway by autocrine signaling, and promote EMT of tumor cells.^246^ It has been revealed that ncRNAs such as miR-98,^247^ miR-193b,^248^ miR-194,^249^ and lncRNA CCAT1^250^ can directly or indirectly regulate IL-10 expression in different tumors to help achieve TIE (Table 6). Therefore, IL-10 may be considered another important target for ncRNA-mediated regulation of TIE. Through the abnormal expression of ncRNAs in tumors, the regulation of IL-10 is likely to be a strategy of tumors to achieve TIE. Therefore, we need to further study these mechanisms, which may become effective targets for cancer treatment in the future.

### VEGF

Vascular endothelial growth factor (VEGF) is an endothelial cell-specific mitogen that is an angiogenesis inducer in a variety of in vivo models and has important physiological functions.^251^ In addition, plenty of evidence shows that VEGF is also an important regulator of pathological angiogenesis. In situ hybridization studies have shown that VEGF mRNA is expressed in most human tumors and can promote tumor progression and help tumors achieve TIE.^252^ Mechanistically, VEGF promotes the proliferation of immunosuppressive cells, inhibits the recruitment of T cells in the TME, and promotes the exhaustion of T cells, thus promoting TIE.^252^ VEGF can also act as a chemokine to recruit Tregs into the TME, thereby affecting the antitumor immune response of immune cells.^253^ In many tumors, it has been found that some abnormally expressed ncRNAs can directly or indirectly regulate the expression of VEGF to promote the development of tumors (Table 6). For example, miR-638,^254^ miR-503,^255^ miR-497,^256^ miR-203,^257^ miR-200,^258^ miR-195,^259^ miR-190,^260^ miR-126,^261,262^ miR-93,^263,264^ miR-29b,^265^ miR-20,^266^ lncRNA TDRG1^267^ and lncRNA HOTAIR^268^ directly up/downregulate VEGF expression in various tumors. In contrast, miR-22,^78^ miR-107,^269^ miR-519c,^270^ miR-26a,^271^ miR-145,^272^ lncRNA H19,^273^ and lncRNA GAS5^274^ were found indirectly to up/downregulate VEGF expression in different tumors. Therefore, similar to TGF-β, IL-6 and IL-10, tumors are likely to utilize the immunosuppressive function of VEGF through ncRNAs to finally achieve TIE. This is likely to become another target of antitumor immunotherapy.

## Tumor exosomes (TEX) and TIE

Exosomes are extracellular vesicles 40–150 nm in diameter that participate in intercellular communication. TEX can transport various ncRNAs and support the associated immunosuppressive functions.^12,275^ Currently, ncRNAs transported by exosomes from various tumor cells have been reported to be associated with immunosuppression and TIE (Table 7). For example, in HCC, miR-23a in TEX can inhibit the expression of PTEN and upregulate PD-L1 expression in macrophages, decrease antitumor immunity and promote TIE.^276^ Furthermore, miR-23a derived from hypoxic TEX can also act as an immunosuppressive factor that directly targets CD107 in NK cells, thus inhibiting the killing function of NK cells on tumors.^277^ Similarly, miR-24-3p, miR-891a, miR-106a-5p, miR-20a-5p, and miR-1980 in nasopharyngeal carcinoma-derived TEX were also found to promote immunosuppression and TIE. These miRNAs can induce the differentiation of Tregs and cause T cell dysfunction, including dysregulation of proliferation, differentiation, and cytokine secretion, by targeting the downregulation of the MAPK1 and JAK/STAT pathways.^278^ In mouse models of lung cancer and sarcoma, TEX-derived *miR-214* inhibits PTEN and the signals downstream of PTEN (to some extent) in T cells, thereby promoting the differentiation of Tregs and enhancing immunosuppression.^279^ TEX-derived lncRNAs were also found to be associated with immunosuppression and TIE. lncRNA ZFAS1 from gastric cancer-derived TEX can suppress tumor cell apoptosis and promote EMT, which may help gastric cancer to achieve TIE.^280^ TEX isolated from the bladder cancer cell line 5637 show high lncRNA UCA1 expression, which promotes bladder cancer progression by promoting EMT in tumor cells.^281^ In add ition, TEX derived from lung cancer show increased lncRNA MALAT1 expression, which promotes tumor growth and metastasis and inhibits tumor cell apoptosis.^282^ These TEX-derived ncRNAs can promote immunosuppression, inhibit tumor apoptosis and promote tumor EMT through a variety of mechanisms, which are significant driving forces for TIE. This may also be one of the potential causes of immunotherapy tolerance, which needs to be studied in depth.

**Table 7:** ncRNAs transferred by TEX participate in TIE

ncRNAs transferred by TEX	Function	Tumor type	Refs.
miR-24-3p, 891a, 106a-5p, 20a-5p and 1908	Promote T cell dysfunction by downregulating the MAPK1 pathway, thereby inhibiting the antitumor immunity and promote TIE	Nasopharyngeal carcinoma	^278^
*miR-214* and miR-214	Promote the Treg phenotype by inhibiting PTEN in T cells, thereby inhibiting the antitumor immunity and promote TIE	Mouse lung cancer and sarcoma, NSCLC, Multiple myeloma	^279,472,473^
miR-23a	Upregulates PD-L1 expression in macrophages and inhibits NK cells via downregulate CD107, thereby inhibiting the antitumor immunity and promote TIE	HCC, CML	^276,277^
miR-212-3p	Inhibits antigen presentation process by downregulating MHC class II molecules in immature DC cells, thereby inhibiting the antitumor immunity and promote TIE	Pancreatic cancer	^474^
miR-21 and miR-29a	Lead to the M2-type polarization of TAMs and promotes immunosuppression and TIE	NSCLC	^475,476^
lncRNA ZFAS1	Suppresses apoptosis of tumor cells and promotes the process of EMT, thereby promote TIE	Gastric cancer	^280^
lncRNA UCA1	Promotes the process of EMT, thereby promote TIE	Bladder cancer	^281^
lncRNA MALAT1	Promotes tumor growth, metastasis and inhibits tumor cell apoptosis, thereby promote TIE	Lung cancer	^282^

*CML* chronic myeloid leukemia

## Conclusions and perspectives

In this review, we discussed the different TIE mechanisms and summarized the regulatory roles of ncRNAs involved in these mechanisms. Although ncRNAs have been confirmed to be directly associated with TIE, the precise molecular mechanisms underlying such regulation are yet to be elucidated. Immunotherapy often results in low response rates owing to the multiple TIE mechanisms active in tumor cells, and the role of ncRNAs in TIE may be underestimated. Thus, ncRNAs may be considered potential candidates to therapeutically target such TIE mechanisms and are expected to be the key to overcoming the challenges associated with immunotherapy.

Several recent studies have reported the role of ncRNAs in tumor therapy, demonstrating the therapeutic potential of ncRNAs.^283–286^ For example, miR-122 expression is low in liver cancer cells, and delivery of miR-122 in liver tumor cells using LNP-DP1, a cationic lipid nanoparticle formulation, can effectively suppress tumor growth by inhibiting target genes and angiogenesis.^287^ Although the potential of ncRNAs as tumor therapeutic targets has been reported, no study or clinical experiment has reported whether ncRNAs can be considered targets to inhibit TIE. Therefore, considering the key role of ncRNAs in TIE, further research is warranted to explore such therapeutic approaches to improve the efficiency of tumor immunotherapy and reduce the associated side effects. Because ncRNAs are critical in promoting TIE, the potential of ncRNAs as targets for TIE therapy should not be underestimated.

This review summarizes the complex regulatory network of ncRNAs specific to TIE. Some studies provide direct evidence of the involvement of certain ncRNAs in TIE, whereas other studies report observations suggestive of the involvement of certain ncRNAs in the regulation of immune escape-related mechanisms. Although not conclusively investigated, these ncRNAs may be indirectly involved in TIE and can also be explored as potential targets for TIE therapy. Some ncRNAs have both tumorigenic and antitumorigenic properties, and studies involving such ncRNAs may provide new insights into TIE mechanisms and immunotherapy. Further studies verifying the regulatory relationship between ncRNAs and TIE will provide a direction for future studies aimed at developing novel cancer therapeutic approaches. Aside from miRNAs, lncRNAs, and circRNAs, other ncRNAs, such as tRNAs (including their derived tiRNAs and tRFs), rRNAs, and snRNAs, have not been reported to be involved in the regulation of TIE, and further research in this area is essential.
